# Quality, Reliability, and Dissemination of Intrahepatic Cholestasis of Pregnancy Information on Short-Video Platforms in China: A Cross-Sectional Study

**DOI:** 10.7759/cureus.102610

**Published:** 2026-01-30

**Authors:** Jie Chen, Caixia Tian, Limin Chen

**Affiliations:** 1 Obstetrics and Gynecology, Shaoxing University, Shaoxing, CHN; 2 Nursing, Shaoxing University, Shaoxing, CHN

**Keywords:** cross-sectional, global quality score (gqs), intrahepatic cholestasis of pregnancy (icp), social-media, tiktok

## Abstract

Background

Intrahepatic cholestasis of pregnancy (ICP) is a high-risk liver disorder complicating pregnancy. Short-video platforms are a major health information source, yet the quality of ICP-related content is unknown. This study aimed to explore the quality, reliability, and dissemination of ICP-related information online.

Methods

A cross-sectional study was conducted on December 11, 2025. The top 100 videos for the Chinese ICP term from Kwai, Red Notes, and TikTok were screened. Video basic characteristics and engagement metrics were extracted. Quality was assessed using the Global Quality Scale (GQS), a modified Decision-making Information Support Criteria for Evaluating the Reliability of Non-randomised Studies (mDISCERN), Journal of the American Medical Association (JAMA) Benchmark criteria, and a Content Completeness Score (CCS). Non-parametric data were summarized using medians and interquartile ranges (IQRs). Group comparisons were conducted with the Kruskal-Wallis test, and correlations were assessed via Spearman's correlation analysis. Statistical significance was set at p < 0.05, with analyses performed using IBM SPSS 30.0 (IBM Corp., Armonk, NY) and a few online platforms.

Results

A total of 174 videos were included for systematic analyses. Video quality was moderate (GQS median 3.00) and varied across three platforms. Content completeness was suboptimal (CCS median 5.00). Videos from healthcare professionals scored higher. User engagement metrics (likes, comments) were significantly higher on TikTok (ByteDance Ltd., Beijing, China) but showed negligible or weak correlations with quality scores across all platforms.

Conclusion

The quality of ICP information on short-video platforms is inconsistent and often incomplete, despite high social engagement. Professional sources are more reliable, but significant informational gaps persist. This highlights a public health need for improved platform governance, professional content creation, and enhanced digital health literacy for pregnant women.

## Introduction

Intrahepatic cholestasis of pregnancy (ICP) is the most common pregnancy-related liver disorder, typically presenting in the late trimester with pruritus and elevated serum bile acids [1]. It is associated with increased risks of preterm birth, meconium-stained amniotic fluid, and stillbirth, particularly in severe cases [1,2]. Its pathogenesis is multifactorial, involving genetic susceptibility, hormonal influences, and environmental factors, and diagnostic criteria and management strategies remain heterogeneous [1]. Given the potential severity of fetal complications and the complexity of clinical decision-making, timely access to accurate, reliable, and evidence-based information on ICP is crucial for pregnant women and their families [1,3].

With the rapid expansion of internet access, short-video platforms have now become an increasingly important source of health information for the public, particularly among women of reproductive age [4-6]. China has the world’s largest population of internet users, accounting for a substantial proportion of global short-video consumers. Therefore, patterns observed on Chinese platforms may have broad relevance beyond a single national context. Compared with traditional text-based resources, short videos offer visually engaging and easily digestible information, which may enhance health communication efficiency. Nowadays, Douyin (Chinese version of TikTok, ByteDance Ltd., Beijing, China), Bilibili (Bilibili Inc., Shanghai, China), and Kwai (Kuaishou Technology, Beijing, China) have become major short video platforms in China [7-9]. However, the accuracy, completeness, and reliability of medical information disseminated through these platforms remain highly variable.

Previous studies evaluating short-video content related to a range of diseases, and the results demonstrated suboptimal overall quality and low reliability [7,10,11]. Nevertheless, existing evidence is largely derived from studies on oncology and chronic medical conditions, leaving pregnancy-related disorders underexplored [6,12-14]. Despite the clinical significance of ICP and its implications for maternal and fetal outcomes, no systematic evaluation has been conducted to assess the quality and reliability of ICP-related information on short-video platforms. Given the increasing reliance of pregnant women on the internet for disease-relevant knowledge, this gap is concerning. Therefore, this cross-sectional study aims to evaluate and compare the content completeness, quality, and reliability of ICP-related short videos across three major Chinese platforms (Red Notes (Xingyin Information Technology Co., Ltd., Shanghai, China), Kwai, and TikTok). We hypothesize that the overall quality of ICP-related videos will be suboptimal and that content from healthcare professionals will be more reliable than that from non-professional uploaders. The findings will be essential for identifying misinformation, guiding platform governance, and improving digital health education for pregnancy-related liver diseases.

## Materials and methods

Video selection and data extraction

A cross-sectional study was conducted to identify and analyze short videos related to Intrahepatic cholestasis of pregnancy (ICP) on three popular Chinese social media platforms: Douyin (Chinese TikTok: www.douyin.com), Red Notes (www.xiaohongshu.com), and Kwai (http://www.kuaishou.com). The Chinese term "妊娠期肝内胆汁淤积症" for " Intrahepatic cholestasis of pregnancy (ICP)" was used as the sole search term across all websites. To mitigate the influence of personalized algorithms, all searches were executed in a standardized manner using the private browsing mode of a web browser without any user login, thereby ensuring access was in a default "visitor mode." The default comprehensive sorting algorithm on each platform was used to simulate the experience of a typical first-time user. All data collection was completed on December 11, 2025, to maintain temporal consistency and avoid dynamic fluctuations in platform content. The initial sample comprised the top 100 videos from the search results on each platform, yielding 300 videos for initial screening.

Eligibility criteria and screening process

Video eligibility was determined through independent review by two authors against predefined criteria. Videos were included if their primary content was directly relevant to ICP, covering aspects such as epidemiology, etiology, symptoms, diagnosis, treatment, or prognosis. Exclusion criteria were applied to remove duplicate videos, content deemed irrelevant or purely promotional, videos shorter than 10 seconds, and videos published within the week prior to data collection. Disagreements during screening were resolved through discussion or adjudication by a third reviewer. Following this screening process, a final sample of eligible videos was established for analysis.

For each included video, a set of characteristics and metrics was extracted. Basic data included the platform, unique URL, publication date, and video duration (converted to seconds). Social engagement metrics: the counts of likes, comments, and collections were recorded as publicly displayed at the time of extraction. Uploaders were classified into three categories: "Specialist" (verified or self-identified licensed medical practitioners such as obstetricians), "Institution" (official accounts of hospitals, universities, or medical societies), and "Individual" (patients, family members, or general users without verifiable medical credentials). This classification was based on profile descriptions, verification badges, and the context of the content. All classifications were agreed upon between the two reviewers before further video quality assessment.

Video quality assessment

The quality, reliability, transparency, and content completeness of included videos were assessed using four standard tools: the Global Quality Scale (GQS), the modified Decision-making Information Support Criteria for Evaluating the Reliability of Non-randomised Studies (mDISCERN) tool, the Journal of the American Medical Association (JAMA) Benchmark criteria, and the Content Completeness Score (CCS). The overall quality was assessed using the GQS, which was rated from 1 (poor quality) to 5 (excellent quality) by the reviewer independently [15]. The reliability of the health information was then evaluated via the mDISCERN tool, which grades a topic in five key domains: 1) clarity of aims, 2) use of reliable sources, 3) balance of information, 4) additional information, and 5) area of uncertainty. Each scored as 0 or 1, yielding a total score between 0 and 5 [16]. Transparency and accountability were assessed via the JAMA criteria, covering four aspects: authorship, attribution, disclosure, and currency; each scored 0 or 1, resulting in a total score from 0 to 4 [17-18]. The comprehensiveness of content was assessed using the CCS, which assesses coverage across six dimensions: epidemiology, etiology, symptoms, diagnosis, treatment, and prognosis. Each dimension is rated as 0 (not mentioned), 1 (partly mentioned), or 2 (fully mentioned). The total score is between 0 and 12 [19]. All assessments were conducted independently by two reviewers (Jie Chen and Limin Chen), both of whom are healthcare professionals with relevant clinical experience. Any disagreement in scores was resolved by the third reviewer (Caixia Tian).

Statistical analysis

Descriptive statistics were used to analyze video characteristics, quality, and reliability metrics. Continuous variables were summarized using medians and interquartile ranges (IQR), while categorical variables were described with frequencies and percentages. The Kruskal-Wallis test was used to compare non-normal distribution continuous variables across three or more platforms or groups. The relationship between non-normally distributed continuous variables was assessed using Spearman correlation analysis. A two-tailed p-value of less than 0.05 was considered statistically significant. All statistical computations were conducted using IBM SPSS 30.0 and the online statistical platforms (www.hiplot.cn, www.zstats.net).

## Results

Video characteristics

**Figure 1:**
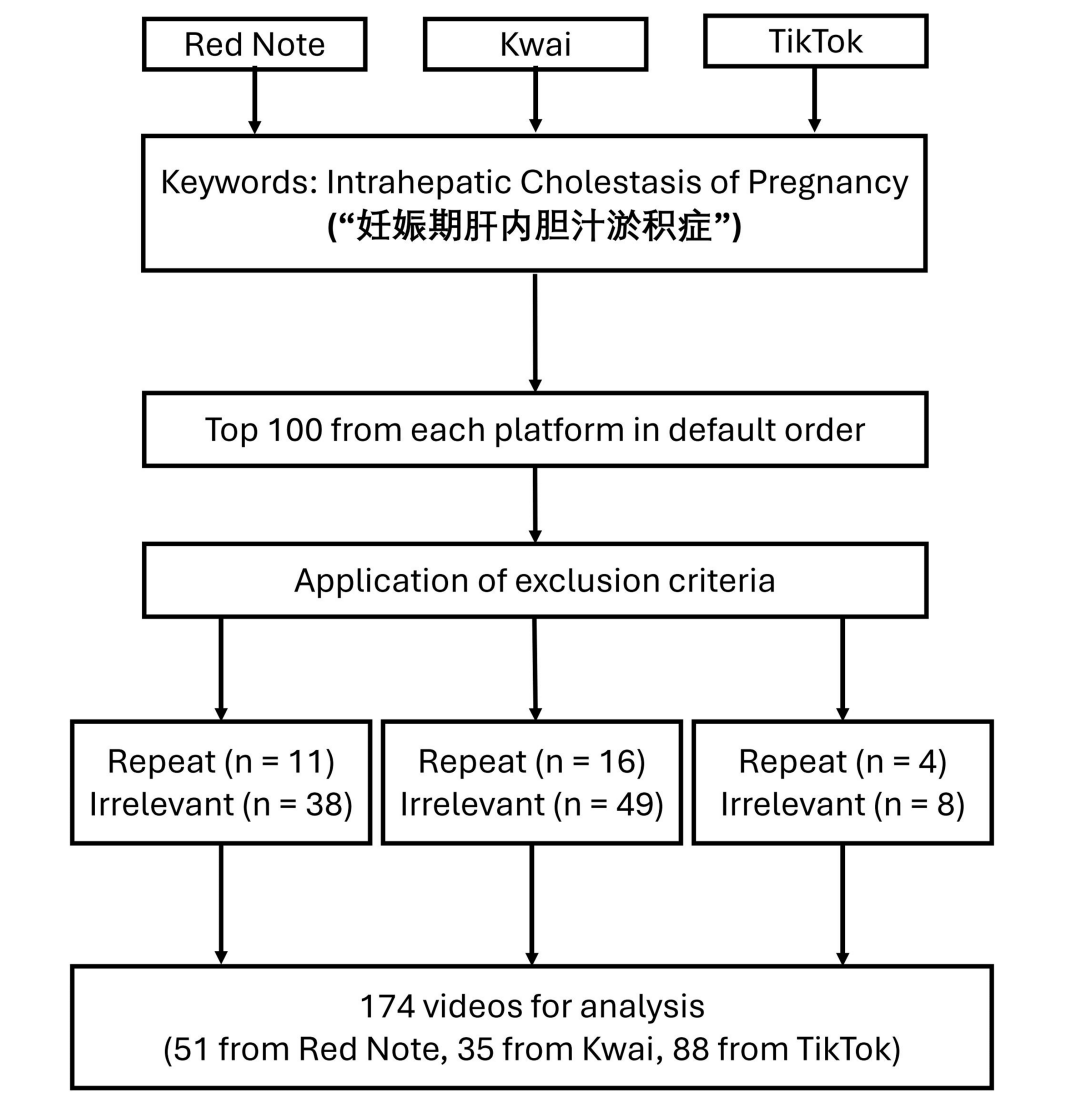
Search strategy, video screening, and selection process.

A total of 174 short videos related to intrahepatic cholestasis of pregnancy (ICP) were included in the final analysis after applying exclusion criteria: Kwai (n = 35), Red Notes (n = 51), TikTok (n = 88) (Figure 1). The platforms differed notably in basic characteristics. Despite similarity in video length, variations were observed in uploader type, quality, and engagement indicators, reflecting distinct platform landscapes and user behaviors (Table 1). Specialist-generated content accounted for a substantial proportion on some platforms, whereas others showed a higher prevalence of user-generated or mixed-source videos. Engagement metrics, including views, likes, comments, and collections, demonstrated wide dispersion across platforms, indicating heterogeneous dissemination patterns and audience responses.

Video sources

**Figure 2:**
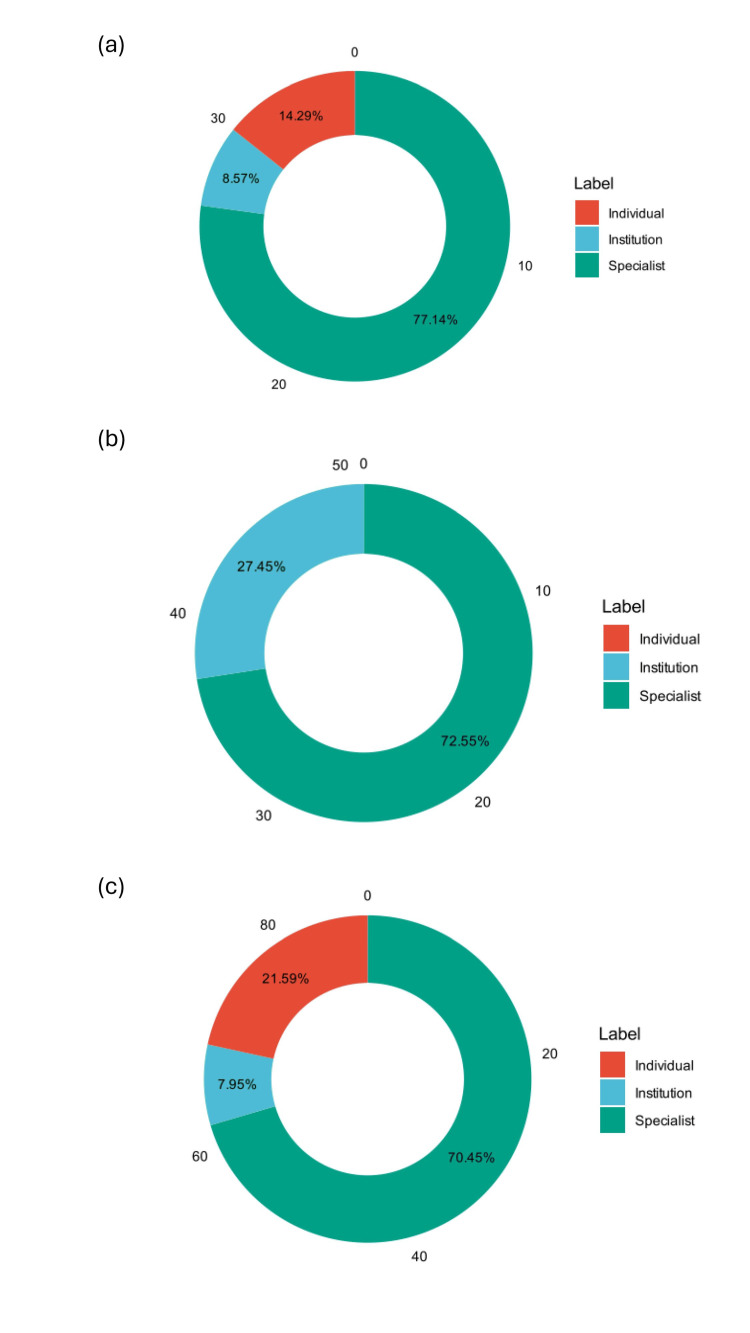
Distribution of uploaders across (a) Kwai, (b) Red Notes, and (c) TikTok.

Regarding sources of videos, the proportions of the three uploaders vary across the three social media platforms. On Kwai, specialists were predominant at 77.14%, followed by individuals at 14.29% and institutions at 8.57%. Red Notes shows a similar trend with specialists at 72.55%, institutions at 27.45%, while individuals did not contribute to this website. TikTok presented a more balanced distribution, with specialists at 70.45%, individuals at 21.59%, and institutions at 7.95%. The data highlight the varying distribution of uploaders across these platforms (Figure 2).

**Table 1:** Baseline information of ICP-related videos across three platforms. ICP, intrahepatic cholestasis of pregnancy

Variables	Kwai (n = 35)	RN (n = 51)	TikTok (n = 88)	p-value
Video length, M (Q₁, Q₃)	56.00 (41.50, 66.00)	63.00 (50.50, 100.50)	58.00 (42.50, 90.25)	0.103
Likes, M (Q₁, Q₃)	98.00 (42.00, 263.00)	9.00 (4.50, 27.00)	338.50 (83.75, 1228.50)	<0.001
Collections, M (Q₁, Q₃)	15.00 (5.00, 60.50)	4.00 (1.00, 14.00)	126.00 (16.25, 588.75)	<0.001
Comments, M (Q₁, Q₃)	17.00 (3.00, 90.00)	2.00 (0.00, 6.50)	86.00 (22.00, 309.25)	<0.001
CCS, M (Q₁, Q₃)	4.00 (3.00, 4.00)	5.00 (4.00, 6.00)	5.00 (4.00, 7.00)	<0.001
GQS, M (Q₁, Q₃)	3.00 (3.00, 3.00)	3.00 (3.00, 3.00)	3.00 (3.00, 4.00)	<0.05
mDISCERN, M (Q₁, Q₃)	2.00 (1.00, 2.00)	2.00 (1.00, 2.00)	2.00 (1.00, 2.00)	0.100
JAMA, M (Q₁, Q₃)	2.00 (2.00, 2.00)	2.00 (2.00, 2.00)	2.00 (2.00, 2.00)	0.299
Uploader, n (%)				<0.001
Individual	5 (14.29)	0 (0.00)	19 (21.59)	-
Institution	3 (8.57)	14 (27.45)	7 (7.95)	-
Specialist	27 (77.14)	37 (72.55)	62 (70.45)	-

Video quality and content

**Figure 3:**
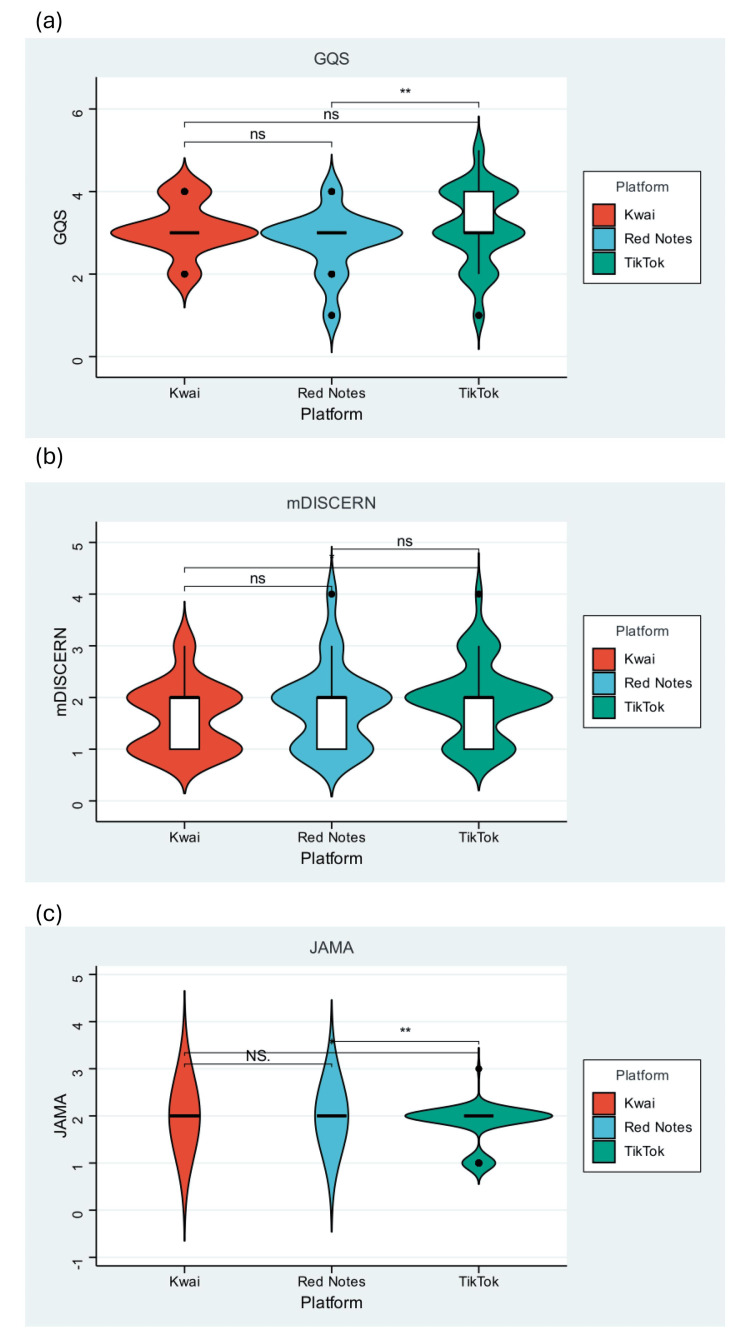
Video quality scores and the quality distribution of videos related to ICP across three platforms: (a) GQS, (b) mDISCERN, (c) JAMA. GQS, Global Quality Scale; ICP, intrahepatic cholestasis of pregnancy; JAMA, Journal of the American Medical Association; mDISCERN, modified Decision-making Information Support Criteria for Evaluating the Reliability of Non-randomised Studies

Overall video quality and content completeness varied substantially across the three platforms. The GQS and CCS were higher in TikTok videos, whereas mDISCERN and JAMA scores were similar across the three websites (Table 1, Figure 3). 

**Figure 4:**
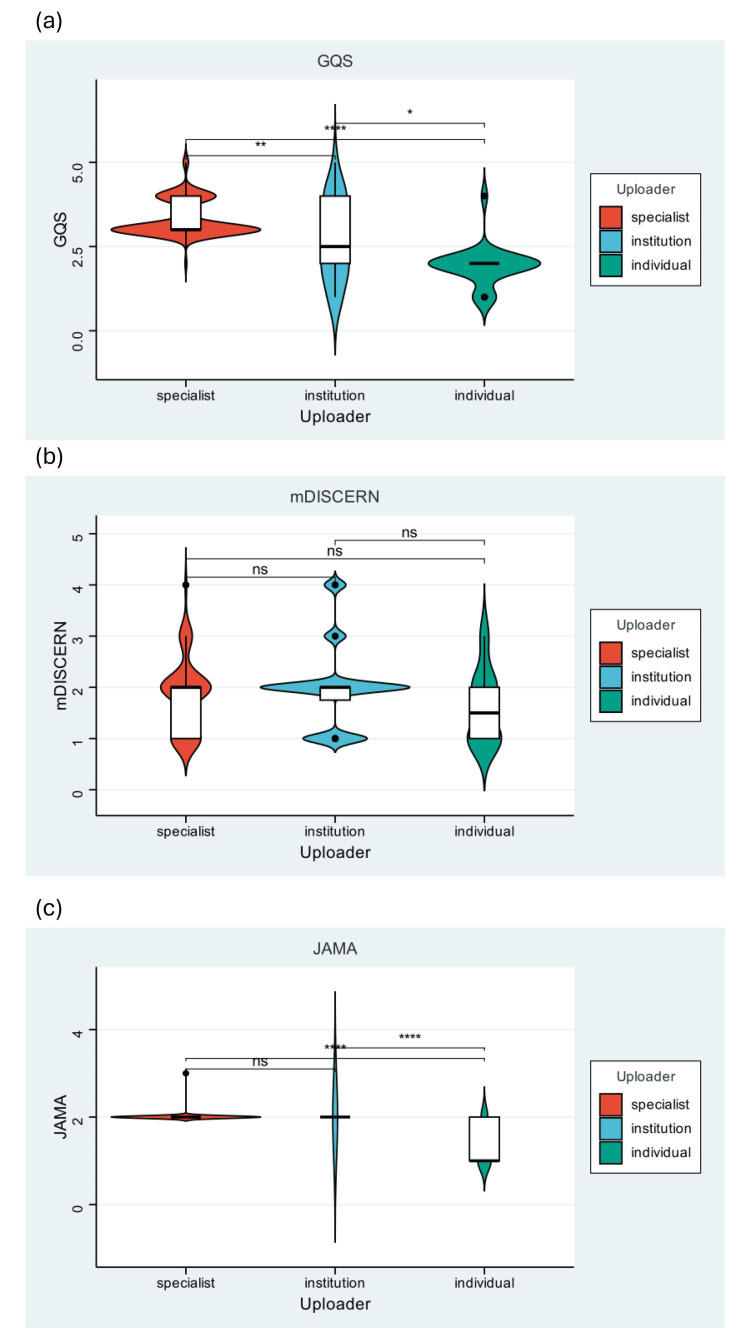
Video quality scores and the quality distribution of videos related to ICP across different uploaders: (a) GQS, (b) mDISCERN, (c) JAMA. GQS, Global Quality Scale; ICP, intrahepatic cholestasis of pregnancy; JAMA, Journal of the American Medical Association; mDISCERN, modified Decision-making Information Support Criteria for Evaluating the Reliability of Non-randomised Studies

Videos uploaded by healthcare professionals generally achieved higher quality and completeness scores than those produced by general users (Figure 4). However, professional authorship did not uniformly guarantee comprehensive content. For example, there was a considerable proportion of professionally created content on TikTok lacking key clinical information, particularly regarding feto-maternal risks and evidence-based management. The overall video quality was suboptimal (GQS median: 3.00, Table 1).

Correlation analysis between video features and quality

**Figure 5:**
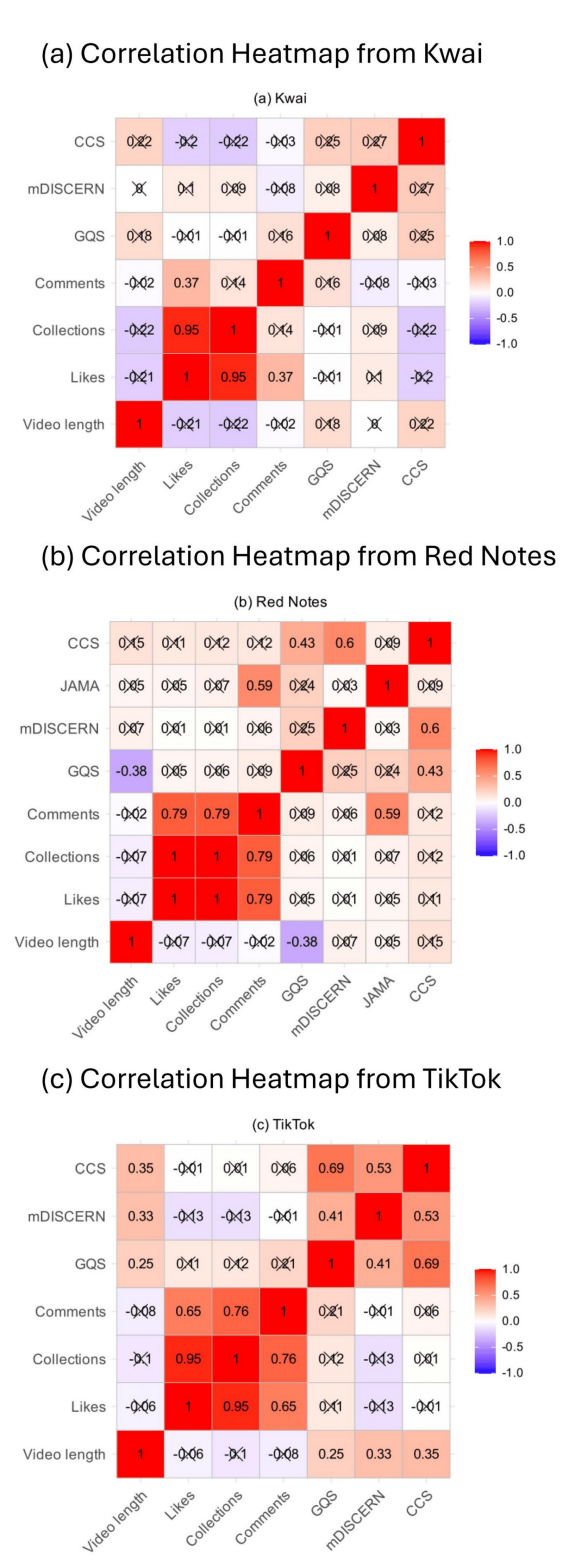
Correlation matrix of video engagement metrics and quality scores on (a) Kwai, (b) Red Notes, and (c) TikTok.

These Spearman correlation analyses indicate how different factors may influence the quality and reliability of ICP information on these platforms (Figure 5). The heatmap for Kwai revealed predominantly negligible or weakly negative correlations between engagement metrics and quality scores. A detailed look identified the strong positive relationship (r = 0.95) between likes and collections. On Red Notes, a similar disconnect between popularity and quality was observed as well. A similar but more prominent correlation (r = 1.0) was found between likes and collections. For TikTok, while most associations remain weak, a moderately positive correlation existed between content completeness and overall quality (r = 0.69). Another strong interconnection was evident (r = 0.76) between collections and comments instead of likes. The platform-specific correlation heatmaps further illustrated heterogeneous interaction patterns between dissemination metrics and content quality. This reflected differences in algorithmic recommendation mechanisms and user engagement behaviors.

## Discussion

This cross-sectional study provides a systematic evaluation of the quality and reliability of ICP-related information on three dominant Chinese short-video platforms. Our findings reveal a landscape of highly variable content quality, with significant proportions of videos lacking comprehensive, evidence-based information crucial for patient understanding and decision-making. These results underscore the risks of using these platforms as health information sources for a potentially high-risk condition like ICP. Our primary finding suggests that the overall information quality is suboptimal. This aligns with prior research assessing health content on short-video platforms for other conditions, such as gestational diabetes mellitus, prostate cancer, and cataract surgery [4,7,10]. The observed median scores on the GQS, mDISCERN, and JAMA criteria indicate that most videos had poor quality, failing to meet standards for reliability, transparency, and balanced information. Taken together, the search algorithms are likely driven by prioritizing social engagement over accuracy, and there was a lack of formal editorial oversight [5,7].

Furthermore, there was a significant association between uploader identity and content quality. Videos created by healthcare professionals or institutions consistently achieved higher GQS and CCS scores. This reinforces findings from studies on cerebral palsy and cardiopulmonary exercise-related videos, where professional sourcing was a strong predictor of information accuracy [5,19]. However, many specialist-created videos still omitted critical details, such as specific fetal monitoring protocols or the indications for early delivery in severe ICP. This "knowledge gap" among some professional creators indicates the need for standardized, evidence-based communication frameworks tailored for the short-video format to ensure brief content covers essential and balanced information. Notably, there were platform-specific variations. Differences in video length, uploader type, and content focus reflect the distinct cultures and user bases of TikTok, Red Notes, and Kwai. This heterogeneity implies that public health interventions and quality improvement efforts must be platform-specific, collaborating with each platform's unique content creators and algorithmic structures. The weak relationship between video duration and quality contradicts a potential assumption that longer videos are inherently more informative. This suggests that content creators should focus on structural clarity and prioritization of key messages rather than simply extending runtime. More importantly, the disconnect between high engagement metrics (likes, comments) and high-quality scores is a major public health concern. It indicates that popularity may be a poor proxy for reliability, potentially amplifying appealing yet inaccurate narratives. This finding echoes studies on tachycardia and pancreatic cancer videos, where viral content was often misleading [11,14]. Platform algorithms that reward engagement without quality safeguards could facilitate the spread of misinformation.

The implications of our findings are multifaceted. For platform regulators, there is a pressing need to develop and implement quality-rating systems or "health information credibility" badges that are algorithmically promoted [20]. Partnerships with medical associations to create certified content hubs could elevate reliable information. For healthcare professionals and institutions, our study highlights both an opportunity and a responsibility [12]. Engaging with these platforms to produce accurate, concise, and engaging content is essential to fill the current quality void [12,21]. For the public, these results emphasize the critical need for improved digital health literacy. Users should be aware of how to judge a certain piece of online health information by checking the uploader's credentials and cross-referencing information with multiple trusted medical sources [22].

This study has several strengths, including that it is the first to explore the quality and reliability of ICP-related content on multiple social media platforms. All data were structurally extracted and analyzed systematically via four validated assessment tools (GQS, mDISCERN, JAMA, CCS). This provides a robust, multi-dimensional evaluation of the quality, reliability, and completeness of the online videos. Additionally, the reviewing process by two independent raters can mitigate bias and misinterpretations.

However, several limitations of this study must be acknowledged. First, the cross-sectional design only captures a single point in time, but the content and platform algorithms are dynamic. Our results only provide a snapshot that may not reflect the platform landscape over time. Second, our search strategy relied on a single, formal Chinese medical term for ICP. While this ensured specificity, it may have excluded relevant layperson-generated content that uses synonymous phrases or colloquial terms, potentially underestimating the volume and varying the quality spectrum of available information. Moreover, the Chinese content may limit the generalization of conclusions to other linguistic social media platforms. Third, by analyzing only the top 100 search results per platform, our sample was inherently shaped by each platform's proprietary sorting algorithm. This approach, while simulating a typical user's experience, may not represent the full breadth of available content, particularly videos with lower visibility but potentially higher quality. Lastly, though this study was independently reviewed by two investigators and further evaluated by inter-rater coefficients, the subjectivity may still remain.

## Conclusions

This study reveals significant inconsistencies and informational gaps in the quality of ICP-related content on three major Chinese short-video platforms. Our findings demonstrated that content from healthcare professionals is generally more reliable, while high social engagement does not correlate with informational accuracy. These results underscore a pressing public health issue: the widespread dissemination of suboptimal health information on platforms heavily used by pregnant women. To mitigate the potential risks identified, concerted and platform-specific action is needed. Future longitudinal studies are also required to assess the real-world impact of such content and to evaluate the effectiveness of interventions, such as credibility badges or professional content hubs, in improving information quality. Ultimately, this study highlights the critical need for improved platform governance, proactive engagement from healthcare professionals as content creators, and enhanced digital health literacy among the public.
